# Meta‐Analysis & Review of Blood Neurofilament Light Performance in Diagnosis of All‐Cause Dementia

**DOI:** 10.1002/alz.083772

**Published:** 2025-01-09

**Authors:** Remington E Schmidt, Yogesh Shah, Broadlawns Medical Center

**Affiliations:** ^1^ Des Moines University, West Des Moines, IA USA; ^2^ Broadlawns Medical Center, Des Moines, IA USA

## Abstract

**Background:**

NfL is a non‐specific biomarker that elevates in the presence of axonal damage. In the blood, it has recently been explored as a potential test in evaluating the presence or absence of dementia.

**Method:**

A systematic review and meta‐analysis of 58 studies examining plasma or serum NfL levels as measured on a single molecule array (SIMOA) platform was used to determine the discriminatory capacity of using NfL to distinguish between healthy controls and patients with dementia.

**Result:**

NfL provides reasonable discriminatory capacity between healthy controls and patients with all‐cause dementia (serum AUC=0.85, plasma AUC=0.75). For a reasonable range of cutoffs, NfL is more specific than it is sensitive. The meta‐analysis demonstrated a roughly 2‐fold increase in NfL levels in dementia patients from cognitively healthy controls (1.84‐fold change plasma, 2.77‐fold change serum).

**Conclusion:**

In the setting of memory care, NfL is likely to be most useful in detecting early dementia in patients presenting with subjective or mild cognitive impairment. As baseline NfL levels significantly change across a person’s lifetime, all NfL levels should be compared to population normals during their interpretation.

